# Abscisic acid induces ectopic outgrowth in epidermal cells through cortical microtubule reorganization in *Arabidopsis thaliana*

**DOI:** 10.1038/srep11364

**Published:** 2015-06-12

**Authors:** Shogo Takatani, Takashi Hirayama, Takashi Hashimoto, Taku Takahashi, Hiroyasu Motose

**Affiliations:** 1 Department of Biological Science, Graduate School of Natural Science and Technology, Okayama University, Okayama 700-8530, Japan; 2 Institute of Plant Science and Resources, Okayama University, Kurashiki 710-0046, Japan; 3 Graduate School of Biological Science, Nara Institute of Science and Technology, Ikoma 630-0192, Japan

## Abstract

Abscisic acid (ABA) regulates seed maturation, germination and various stress responses in plants. The roles of ABA in cellular growth and morphogenesis, however, remain to be explored. Here, we report that ABA induces the ectopic outgrowth of epidermal cells in *Arabidopsis thaliana*. Seedlings of *A. thaliana* germinated and grown in the presence of ABA developed ectopic protrusions in the epidermal cells of hypocotyls, petioles and cotyledons. One protrusion was formed in the middle of each epidermal cell. In the hypocotyl epidermis, two types of cell files are arranged alternately into non-stoma cell files and stoma cell files, ectopic protrusions being restricted to the non-stoma cell files. This suggests the presence of a difference in the degree of sensitivity to ABA or in the capacity of cells to form protrusions between the two cell files. The ectopic outgrowth was suppressed in ABA insensitive mutants, whereas it was enhanced in ABA hypersensitive mutants. Interestingly, ABA-induced ectopic outgrowth was also suppressed in mutants in which microtubule organization was compromised. Furthermore, cortical microtubules were disorganized and depolymerized by the ABA treatment. These results suggest that ABA signaling induces ectopic outgrowth in epidermal cells through microtubule reorganization.

Plant development is deeply dependent on how and where cells grow and divide. Plant cells are generated by oriented cell divisions in the apical meristem and expand directionally in order to form various organs and tissues. Therefore, directional cell expansion and oriented cell division are precisely controlled during plant morphogenesis.

Microtubules play crucial roles during the directional expansion and oriented division of plant cells. Microtubules are composed of αβ-tubulin heterodimer and exhibit highly dynamic self-organizing behavior^1^. The microtubule reorganization is involved in both plant development^2^,^3^ and responses to environmental stimuli^4^. Just after cell division (early interphase), cortical microtubules align longitudinally to suppress cell elongation and are then arranged perpendicularly to allow cells to grow anisotropically^5^. Pharmacological and genetic modifications of microtubules have been shown to affect cell expansion and morphology^1^,^6^. A series of tubulin mutants of *Arabidopsis thaliana* show helical organ growth, which is due to the formation of helical arrays of cortical microtubules^7^. In addition, mutations in genes encoding microtubule-associated proteins (MAPs) cause defects in anisotropic cell growth and cortical microtubule organization^8^,^9^,^10^,^11^,^12^,^13^. Cortical arrays of microtubules, therefore, determine the growth direction of plant cells.

Phytohormones have been shown to regulate cell growth via the rearrangement of the cortical microtubule array^14^. The addition of external auxin and gibberellin promotes the transverse arrangement of cortical microtubules^15^,^16^,^17^, whereas ethylene promotes the longitudinal arrangement of cortical microtubules^18^. A recent study indicates that auxin promotes microtubule isotropy during organ initiation at the shoot apical meristem^19^.

Abscisic acid (ABA) plays essential roles in plant development and adaptation to the environment^20^,^21^,^22^. ABA is associated with seed maturation and dormancy, germination, stomatal closure, and with responses to various stresses such as drought, salt, and cold. ABA mainly promotes stress tolerance through the regulation of gene expression during the period of adaptation to various stressful conditions. Recent molecular and genetic studies revealed the core ABA signaling pathway^22^,^23^,^24^,^25^. ABA binds to the ABA receptor proteins PYrabactin Resistance1/PYR-Like proteins/Regulatory Component of ABA Receptor1 (PYR1/PYLs/RCAR1) and the ABA-receptor complex blocks the activity of clade A protein phosphatase 2C (PP2C) including ABA INSENSITIVE 1 (ABI1). PP2C dephosphorylates and inactivates Sucrose Non-Fermentation 1 (SNF1)-related protein kinase 2 (SnRK2) in the absence of ABA. The ABA-dependent suppression of PP2C results in the activation of SnRK2, which phosphorylates downstream transcriptional regulators such as ABI5 to induce stress-responsive genes.

ABA also modulates microtubule organization and stability. ABA has been shown to increase longitudinal and oblique arrays of cortical microtubules in the epidermal cells of dwarf pea epicotyls^26^,^27^ and in the epidermal and cortex cells of cucumber hypocotyls^28^. Furthermore, ABA and gibberellin have opposite effects on cortical microtubules. ABA treatment suppresses gibberellin-induced transverse microtubule orientation^26^,^27^. ABA increases the cold resistance of cortical microtubules whereas GA decreases it^26^,^27^. Further, ABA has been shown to decrease cortical microtubule abundance and to inhibit seed germination and cell growth in *Coffea arabica*^29^. During stomatal closure, ABA promotes mictotubule depolymerization in guard cells^30^,^31^.

Here we report that the ABA treatment induces ectopic outgrowths in epidermal cells of hypocotyls. The formation of ectopic outgrowths is suppressed in the ABA-insensitive mutants and microtubule-related mutants. ABA treatment promotes cortical microtubule depolymerization and disorganization. Our findings demonstrate that ABA regulates directional cell growth through microtubule reorganization.

## Results

### ABA induces ectopic outgrowth in the epidermis

**Figure 1:**
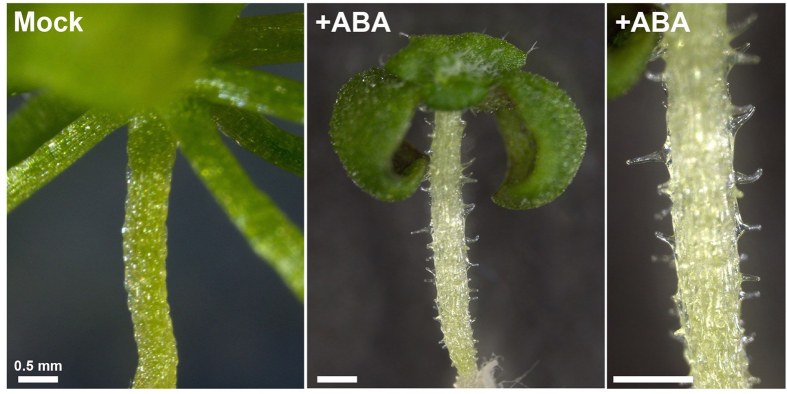
ABA induces ectopic outgrowth in the epidermis. The wild type seedlings were germinated and grown on medium without (Mock) or with 1 μM ABA (+ABA) for 3 weeks.

**Figure 2:**
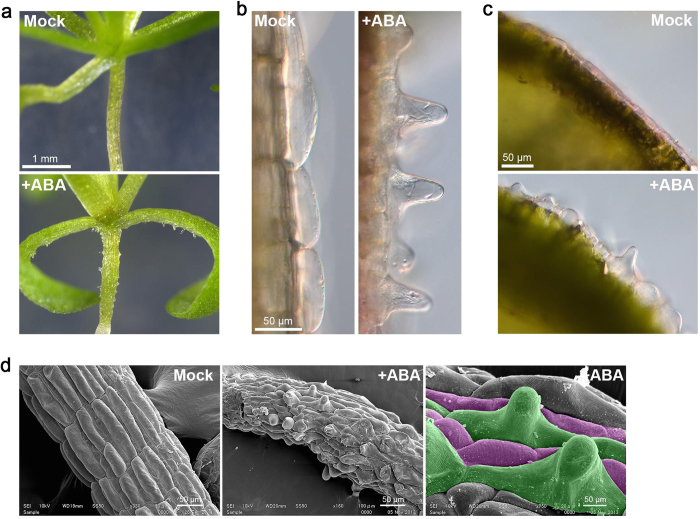
Characterization of ABA-induced ectopic outgrowth in epidermal cells. The wild type seedlings were germinated and grown for 3 weeks on medium without (Mock) or with 1 μM ABA (+ABA). (**a**) Effect of ABA on the morphology of seedlings. Ectopic protrusions were formed on the hypocotyls and petioles. (**b**) Morphology of hypocotyl epidermal cells. Single protrusion was developed in each cell. (**c**) Morphology of adaxial epidermal cells of cotyledons. (**d**) SEM images of hypocotyls. The cell files highlighted in green and magenta indicate non-stomatal cell files and stomatal cell files, respectively.

In previous studies, we found that the *never in mitosis A* (*nimA*)*-related kinase 6* (*nek6*) mutant named *ibo1* exhibits ectopic outgrowth in the epidermal cells of *A. thaliana*^32^,^33^,^34^,^35^. The *nek6* mutants have also been reported to show altered responses to ABA and stresses^36^,^37^. We analyzed the effect of ABA on the seed germination and seedling growth of the wild type and *nek6* mutants. In the course of the experiments, we found that the wild type seedlings germinated and grown in the presence of ABA had ectopic protrusions in the epidermis of hypocotyls, petioles and cotyledons (Figs 1 and 2). This phenotype is reminiscent of that of the *nek6* mutants grown in a medium without ABA. The ectopic protrusions were observed about 10 days after germination and formed in 50–70% of seedlings.

To determine where and how protrusions develop, we conduced detailed observations of ectopic protrusions under a light microscope and a scanning electron microscope (Fig. 2). One protrusion was formed in the middle of each epidermal cell in the hypocotyls and cotyledons (Fig. 2b,c). The cells with ectopic protrusions were vacuolated large cells, in which active streaming of cytoplasmic strands was observed. Hypocotyls of *A. thaliana* have two kinds of epidermal cell files that are arranged alternately: the non-stoma large cell file and the stoma cell file^38^,^39^,^40^. Ectopic protrusions were formed in the non-stoma large cell files (Fig. 2d). This suggests that sensitivity to ABA is different between the two cell files and/or that the non-stoma cell files have the capacity to form protrusions.

Because ABA mediates stress responses, other stress treatments were expected to induce ectopic outgrowth in the epidermal cells. Therefore, we examined the effect of salt stress on epidermal cell morphology (Supplementary Fig. S1). Seedlings under salt stress did not form protrusions in the epidermal cells, suggesting that ectopic outgrowth is caused by the effect of ABA rather than the stress response.

To examine the effect of enhanced cell elongation on ectopic outgrowth, seedlings were germinated and grown on medium containing ABA under dark conditions. The epidermal cells of etiolated seedlings did not develop ectopic protrusions even in the presence of ABA (Supplementary Fig. S2). This result indicates that in the presence of ABA, the promotion of cell elongation counteracts ectopic outgrowth.

To analyze when cells require ABA to form ectopic protrusions, seedlings germinated on the medium without ABA were transferred to the ABA-containing medium. After germination and growth of seedlings for 3 days on the medium without ABA, and then transferred to the ABA-containing medium, the seedlings did not produce ectopic protrusions (Supplementary Fig. S3). This implies that ABA must be present during both germination and the early phase of seedling growth for ectopic protrusions to form.

### ABA signaling mediates ectopic outgrowth

**Figure 3:**
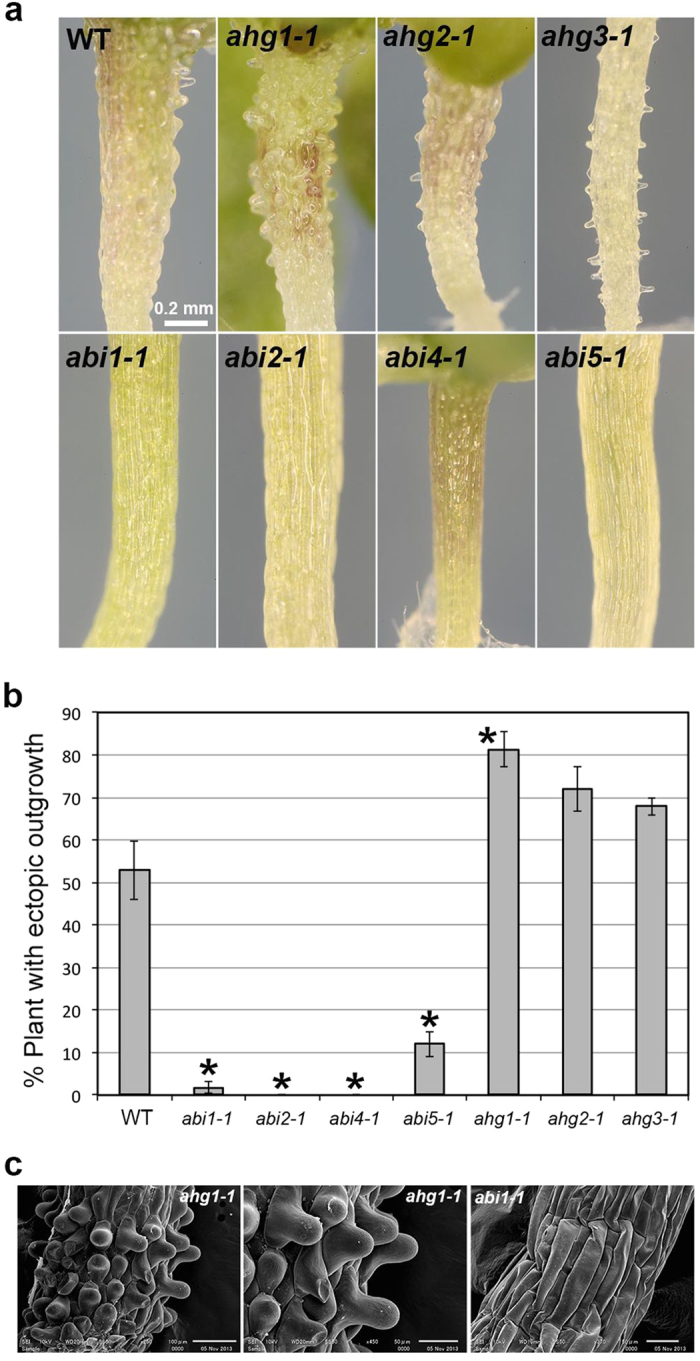
Effect of ABA on the morphology of hypocotyl epidermal cells in ABA-related mutants. (**a**) Morphology of hypocotyls in the wild type (WT) and ABA-related mutants grown for 3 weeks in the presence of 1 μM ABA (ABA). The *abi* mutants showed the suppression of ectopic outgrowth whereas the *ahg* mutants showed enhanced outgrowth. (**b**) Quantification of ectopic outgrowth in the wild type (WT), *abi* mutants and *ahg* mutants. Data are displayed as averages ± SEM of 3 independent experiments. Asterisks indicate significant differences from the value in the wild type (*P* < 0.001) according to Fisher’s exact probability test in *abi1*, *abi2* and *abi4* and according to the chi-square test for the difference between two proportions in *abi5*, *ahg1*, *ahg2* and *ahg3* (*abi1-1*, *P* = 1.7 × 10^−14^; *abi2-1*, *P* = 1.4 × 10^−14^; *abi4-1*, *P* = 2.7 × 10^−16^; *abi5-1*, *P* = 5.0 × 10^−8^; *ahg1-1*, *P* = 3.9 × 10^−6^; *ahg2-1*, *P* = 0.0027; *ahg3-1*, *P* = 0.034). (**c**) SEM images of hypocotyls of *ahg1-1* and *abi1-1* grown for 3 weeks in the presence of 1 μM ABA.

Next, we analyzed the effect of ABA on epidermal morphology in *aba insensitive* (*abi*) mutants. In the *abi1*, *abi2* and *abi4* mutants, epidermal cell morphology was not affected by the presence of ABA and ectopic protrusions were rarely observed (Fig. 3). In the *abi5* mutant, the formation of ectopic protrusions was suppressed compared to in the wild type, but a small number of ectopic protrusions still formed. These results show that the core ABA-signaling components, *ABI1*, *ABI2*, *ABI4* and *ABI5* are involved in ectopic outgrowth formation.

Next, we analyzed the effect of ABA on epidermal cells in *aba hypersensitive germination* (*ahg*) mutants, which show a hypersensitivity to ABA during germination and early seedling growth^41^,^42^,^43^,^44^,^45^,^46^. The *ahg1-1*, *ahg2-1* and *ahg3-1* mutants grown in the presence of ABA exhibited enhanced ectopic outgrowth and produced larger numbers of protrusions than the wild type (Fig. 3a,b). The *ahg3-1* mutant exhibited a mild phenotype compared to the *ahg1-1* and *ahg2-1* mutants. Ectopic outgrowth was promoted in the *ahg* mutants but was also restricted to the non-stoma cell files as in the wild type (Fig. 3c). These results demonstrated that *AHG1*, *AHG2* and *AHG3* genes are required for the suppression of ABA-induced ectopic outgrowth.

### Cortical microtubules are involved in ABA-induced ectopic outgrowth

**Figure 4:**
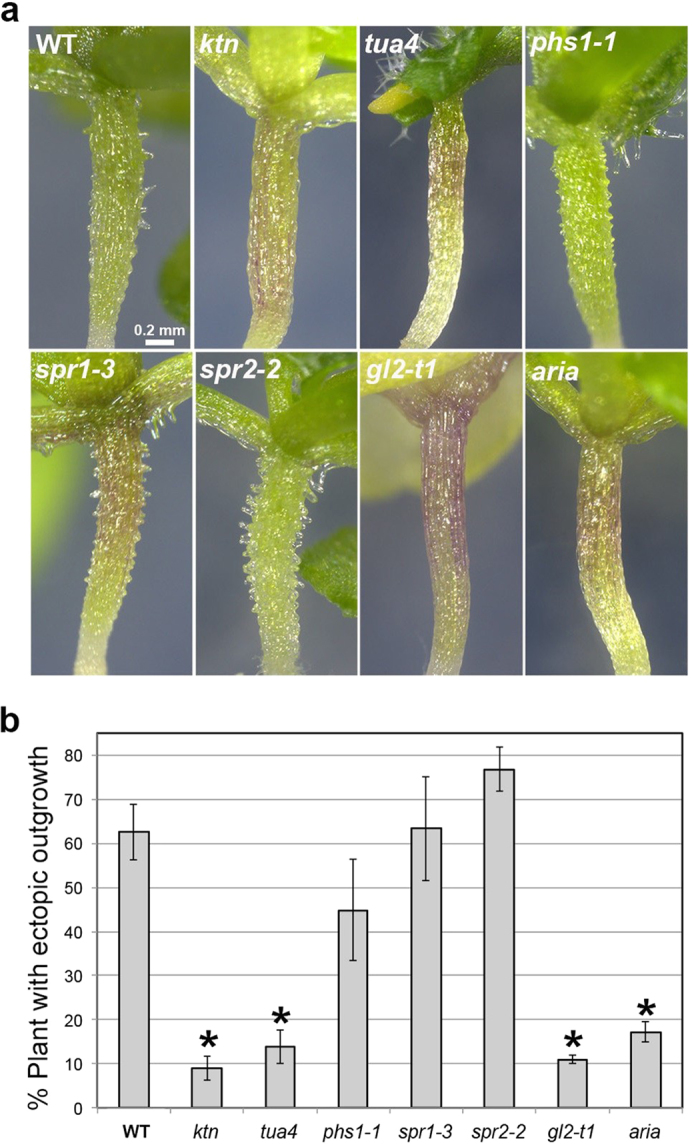
Effect of ABA on the morphology of hypocotyl epidermal cells in various mutants. (**a**) Morphology of hypocotyls in the wild type (WT) and mutants grown for 3 weeks in the presence of 1 μM ABA. The *ktn1*, *tua4*, *gl2* and *aria* mutants exhibited reduced ectopic outgrowth whereas the *phs1*, *spr1* and *spr2* mutants exhibited ectopic outgrowth. (**b**) Quantification of ectopic outgrowth in the wild type (WT) and various mutants. Data are displayed as averages ± SEM of 3 independent experiments. Asterisks indicate significant differences from the value in the wild type (*P* < 0.001) according to the chi-square test for the difference between two proportions (*ktn1*, *P* = 3.0 × 10^−16^; *tua4*, *P* = 3.1 × 10^−15^; *phs1-1*, *P* = 0.0044; *spr1-3*, *P* = 0.33; *spr2-2*, *P* = 0.0018; *gl2*, *P* = 3.5 × 10^−9^; *aria*, *P* = 3.4 × 10^−11^).

To examine the involvement of microtubules in ectopic outgrowth formation, we analyzed the effect of ABA on epidermal cells of the microtubule-related mutants including *katanin1* mutant (*ktn1 P393S*), *α-tubulin 4* mutant (*tua4 S178δ*), *propyzamide hypersensitive 1-1d* (*phs1-1d*), *spiral1-3* (*spr1-3*) and *spr2-2* (Fig. 4). Katanin is an ATP-dependent microtubule-severing enzyme composed of p60 ATPase and the p80 regulatory subunit^47^,^48^. The *ktn1* mutant carries a mutation in the p60 subunit of KATANIN1 (KTN1). SPR1 and SPR2 are plant-specific MAPs involved in directional cell expansion and microtubule dynamics^8^,^9^,^10^,^11^,^12^,^13^. Propyzamide HiperSensitive 1 (PHS1) is a chimeric protein harboring α-tubulin kinase domain and mitogen-activated protein kinase (MAPK) phosphatase domain^49^,^50^. PHS1 prevents microtubule polymerization by α-tubulin phosphorylation in response to osmotic stress^50^. In the dominant *phs1-1d* mutant, the kinase domain is partially activated and cortical microtubules are destabilized even in the absence of stress. In the *ktn1* and *tua4* mutants, the formation of ectopic protrusions was suppressed compared to that of the wild type. In contrast, ectopic outgrowth was not significantly affected in the *spr1-3* and *phs1-1* mutants. In *spr2-2*, protrusion formation was slightly increased and the number of protrusions per plant seemed to be higher.

**Figure 5:**
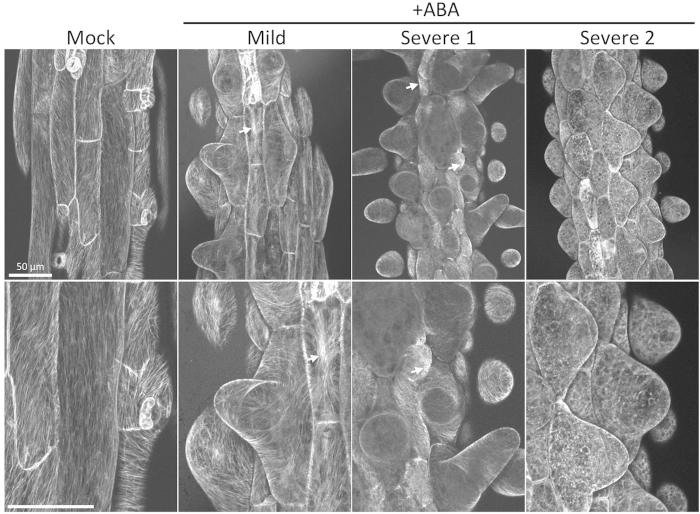
Effect of ABA on cortical microtubules. Z-stack images of hypocotyl epidermal cells in GFP-TUB6 plants grown for 2 weeks on medium without (Mock) or with 1 μM ABA (+ABA). The phenotypes were divided into two groups: mild plants (Mild) and severe plants (Severe 1 and 2). “Severe 2” indicates very severe phenotype. Lower panels are enlarged images of upper panels. Arrows indicate microtubule bundling.

**Figure 6:**
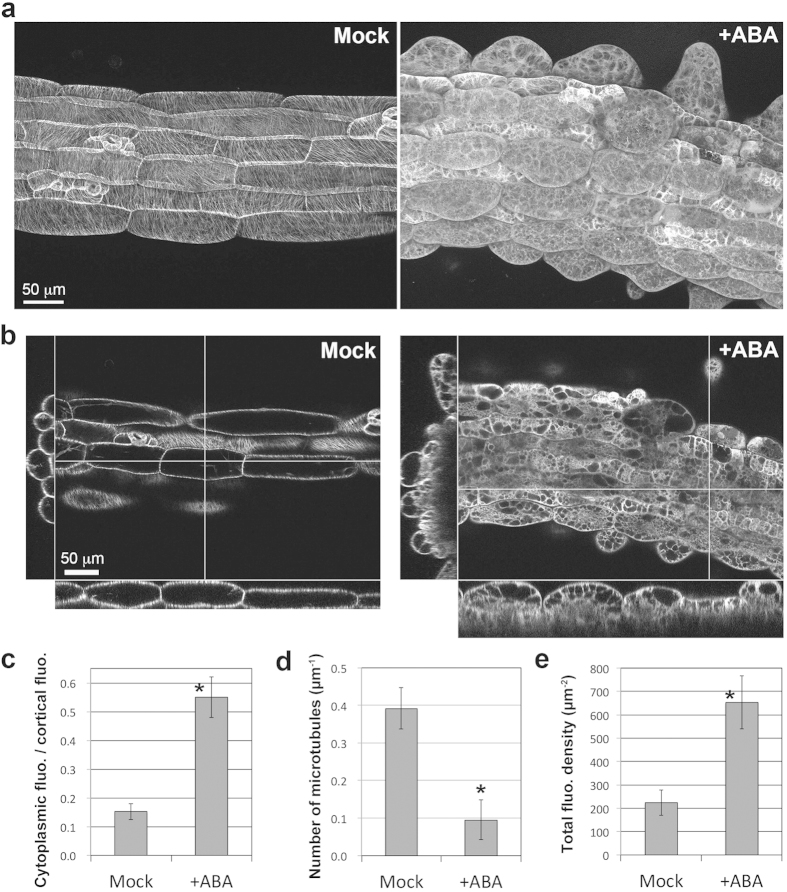
ABA-induced depolymerization of cortical microtubules. (**a**) Z-stack images of hypocotyl epidermal cells of GFP-TUB6 plants grown for 2 weeks on medium without (Mock) or with 1 μM ABA (+ABA). This ABA-treated plant showed severe phenotype and extensive cortical microtubule depolymerization. (**b**) Orthogonal images along the optical axis (x, y) and xy-plane. (**c**) Quantification of the ratio of GFP-TUB6 fluorescence in the cortical region to the fluorescence in the cytoplasm in non-stoma cell files. Data are displayed as averages ± SD (n = 10 cells from 3 plants). (**d**) Quantification of the number of cortical microtubules without (Mock) or with 1 μM ABA (+ABA). Data are displayed as averages ± SD (n = 24 cells from 5 plants). (**e**) Quantification of the total fluorescence density of GFP-TUB6 without (Mock) or with 1 μM ABA (+ABA). Data are displayed as averages ± SD (n = 10 cells from 3 plants). Asterisks in (**c**), (**d**) and (**e**) indicate significant differences from the values in the mock treatment (Mann-Whitney *U* test, *P* = 0.0000054 in (**c**), *P* = 1.4 × 10^−9^ in (**d**), *P* = 0.0000054 in (**e**)).

To analyze the effect of ABA on cortical microtubules, we used GFP-TUB6 lines, which express β-tubulin 6 (TUB6) fused with GFP under the Cauliflower Mosaic Virus (CaMV) 35S constitutive promoter^51^. Cortical microtubules in the hypocotyls and leaves were labeled with GFP-TUB6. The cortical microtubules of the control plants were observed as the clear filaments arranged in parallel arrays transverse, oblique or longitudinal to the hypocotyl axis (Figs 5 and 6, n = 8 plants), whereas cortical microtubules of ABA-treated seedlings were disorganized and depolymerized (n = 20 plants). The phenotypes of ABA-treated seedlings were divided into two groups according to microtubule organization and depolymerization: a mild phenotype and a severe phenotype. In the mild phenotype plants (n = 4/20, 20%), cortical microtubules were remarkably disorganized and slightly depolymerized (indicated as Mild in Fig. 5). The cortical microtubules were curved, whorled and arranged perpendicular to the direction of ectopic outgrowth (Fig. 5). Some cortical microtubules were bundled especially in stoma cell files. These phenotypes partially resemble those seen in the *nek6* mutants. In the severe phenotype plants (n = 14/20, 70%), cortical microtubules were significantly depolymerized and reorganized (Severe 1 and 2 in Fig. 5, +ABA in Fig. 6a,b). The depolymerization of cortical microtubules was confirmed by an increase in cytoplasmic fluorescence (Fig. 6c) and a decrease in cortical microtubule numbers in ABA-treated non-stomatal cell files (Fig. 6d). The total fluorescence of GFP-TUB6 was higher in ABA-treated cells (Fig. 6e), suggesting that the microtubule depolymerization is not due to a tubulin decrease. In the most severely affected plants, the cortical microtubules became obscure, and the cytoplasmic fluorescence was particularly obvious. These severe phenotypes are not observed in the case of *nek6* mutants. Only two plants (n = 2/20, 10%) showed neither ectopic protrusions nor defects in the cortical microtubules. These results demonstrate that ABA induces the ectopic outgrowth of epidermal cells via cortical microtubule depolymerization and reorganization.

Next, we analyzed whether ABA promotes microtubule depolymerization and deformation during a short-term treatment. The GFP-TUB6 seedlings grown in medium without ABA were soaked in ABA-containing liquid medium and observed via confocal microscopy. The ABA treatment promoted the longitudinal orientation of cortical microtubules and stomatal closure, but did not induce the deformation and depolymerization of cortical microtubules (Supplemental Figure S4).

### Effect of a microtubule-depolymerizing drug on ectopic outgrowth

**Figure 7:**
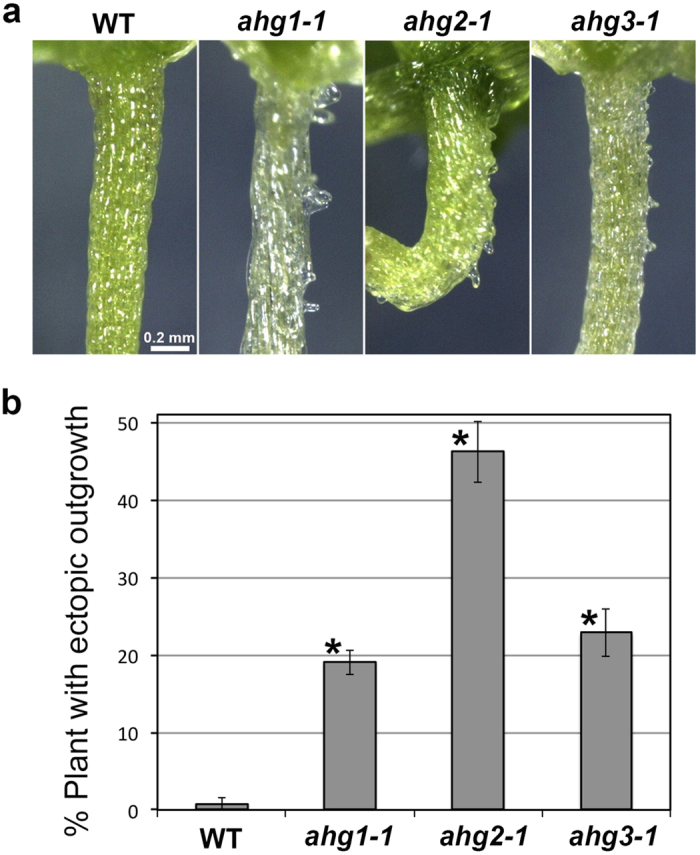
Propyzamide induces ectopic outgrowth in epidermal cells of the *ahg* mutants. (**a**) Morphology of hypocotyls in the wild type (WT) and the *ahg* mutants germinated and grown for 2 weeks in the presence of 3 μM propyzamide. The *ahg* mutants exhibited ectopic outgrowth whereas the wild type did not. (**b**) Quantification of ectopic outgrowth in the wild type (WT) and the *ahg* mutants grown with 3 μM propyzamide. Data are displayed as averages ± SEM of 4 independent experiments. Asterisks indicate significant differences from the value in the wild type (*P* < 0.001) according to Fisher’s exact probability test (*ahg1-1*, *P* = 1.6 × 10^−9^; *ahg2-1*, *P* = 2.6 × 10^−22^; *ahg3-1*, *P* = 2.7 × 10^−10^).

To confirm that ABA induces ectopic outgrowth through microtubule depolymerization, we analyzed effect of propyzamide, a microtubule-depolymerizing drug, on the *ahg* mutants. Because *ahg* mutants have high sensitivity to ABA, propyzamide itself could induce ectopic outgrowth in the absence of exogenous ABA. In fact, addition of propyzamide remarkably induced ectopic outgrowth in the *ahg* mutants but not in the wild type (Fig. 7). The effect of propyzamide was most conspicuous in the *ahg2-1* mutant, which accumulates more endogenous ABA than the wild type^42^. This result suggests that the *ahg* mutants exhibit ectopic outgrowth in response to endogenous ABA when microtubules are destabilized.

Next, we examined whether the addition of propyzamide enhances ABA-induced ectopic outgrowth or not. Addition of propyzamide did not affect ABA-induced ectopic outgrowth (Supplemental Figure S5). This might be due to that strong effect of ABA masks the effect of propyzamide on ectopic outgrowth. Because ABA remarkably depolymerizes microtubules, microtubule depolymerization could not be further enhanced by propyzamide.

### Other factors involved in ectopic outgrowth

The homeodomain transcription factor GLABRA2 (GL2) is required for the alternating cell file organization in hypocotyls^39^,^40^. The *gl2* mutant is defective in the differentiation of non-stomatal cell files. Because ABA induces ectopic outgrowth in the non-stomatal cell files, *gl2* mutation is expected to affect ABA-induced ectopic outgrowth. Therefore, we analyzed the effect of ABA on the hypocotyl epidermis of the *gl2* mutant. The *gl2-t1* mutant used in this study is a T-DNA insertion null allele and have been shown not to express transcripts for *GL2*^52^. The *gl2* mutant showed suppressed protrusion formation (Fig. 4). This result showed that GL2-dependent alternate cell file organization is required for the production of ABA-induced ectopic outgrowths.

ARIA (Armadillo repeat protein interacting with ABF2) is involved in the ABA response, through its interaction with the transcription factor ABF2/AREB1 (ABSCISIC ACID RESPONSIVE ELEMENTS-BINDING FACTOR2)^53^ and also with NEK6^36^. As described above, *nek6* mutants formed ectopic protrusions in their epidermal cells, which are similar to the protrusions formed in the presence of ABA. Therefore, we analyzed the effect of ABA on the epidermis of the *aria* mutant (Fig. 4). The *aria* mutant demonstrated a suppression of ectopic outgrowths, suggesting that ARIA is involved in ABA-dependent ectopic outgrowth formation.

## Discussion

ABA is an essential phytohormone regulating seed maturation, germination, stomatal closure and various stress responses. However, the effect of ABA on cellular growth and morphogenesis has not yet been characterized in detail. Here, we found that epidermal cells developed ectopic protrusions in seedlings germinated and grown in the presence of ABA. This effect is specific to ABA and was not observed in the salt treatment. Because ABA-insensitive mutants including *abi1*, *abi2*, *abi4* and *abi5* exhibited the suppression of protrusion formation, ABA signaling must be required for the ectopic outgrowth of epidermal cells to occur. This is confirmed by the observation that protrusion formation was promoted in the ABA-hypersensitive mutants, including *ahg1*, *ahg2* and *ahg3* and suppressed in ABA less-sensitive mutant *aria*. ABI1 and ABI2 are two major PP2Cs that function as negative regulators of ABA signaling. AHG1 and AHG3 belong to a clade A PP2C, which includes ABI1 and ABI2, and negatively regulate ABA signaling, mainly during seed maturation, germination, and early seedling development^43^,^44^. Therefore, ABA-mediated ectopic outgrowth formation might be suppressed by multiple PP2Cs including ABI1, ABI2, AHG1 and AHG3. In addition, the specific function of AHG1 and AHG3 in seed germination and early growth is well correlated with the requirement of ABA during germination and the early phase of seedling growth for ectopic outgrowth to occur. *AHG2* encodes the polyA-specific ribonuclease (PARN) that modulates ABA and salicylic acid signaling via mitochondrial RNA metabolism^42^,^45^,^46^. The PP2Cs and AHG2-dependent pathway might be coordinately involved in ABA-dependent ectopic outgrowth in epidermal cells.

Ectopic protrusions were observed only in non-stomatal cell files (Figs 2 and 3). This observation correlates well with the suppression of ectopic outgrowth in the *gl2* mutant, which exhibits a defect in the formation of alternate cell files (specifically, a defect in non-stomatal cell files). GL2 might be indirectly required for ectopic outgrowth via epidermal cell differentiation. The cells within the non-stomatal files could be more sensitive to the effect of ABA, or these cells could have some intrinsic property that allows for the formation of outgrowths and that is not found in the other lineage.

Our results suggest that ABA affects cell expansion and morphogenesis through microtubule organization. Among five microtubule-related mutants, *ktn1* and *tua4* mutants showed a remarkable suppression of ectopic outgrowth (Fig. 4). Furthermore, cortical microtubules were disorganized and depolymerized in the presence of ABA (Figs 5 and 6). In addition, propyzamide, a microtubule-depolymerizing drug, induced ectopic outgrowth in the ABA-hypersensitive *ahg* mutants (Fig. 7). These results strongly support the conclusion that ABA induces ectopic outgrowth through microtubule depolymerization.

KTN1 has been shown to regulate anisotropic growth and microtubule organization^48^. Recent analysis revealed that KTN1 is a key regulator for microtubule reorganization and the dynamics of meristem development and environmental response^3^,^4^. KTN1 releases newly branched microtubules from its mother microtubules^54^ and generates growing plus ends by severing microtubules at microtubule crossover sites^4^. The latter mechanism is essential for microtubule rearrangement during phototropism^4^. The katanin-dependent microtubule rearrangement might be involved in ABA-dependent ectopic outgrowth.

The involvement of microtubules in the formation of ABA-dependent ectopic outgrowths is correlated with the fact that several microtubule-related mutants and transgenic plants show ectopic outgrowth in their epidermal cells: *spr1* mutant grown at the low temperature^8^, *nek6/ibo1* mutants^32^,^33^,^34^,^35^ and overexpressor of Basic Proline-rich Protein 1 (BPP1)^55^. Interestingly, these mutants and transgenic plants exhibit a decrease in microtubule dynamics and an increase in microtubule stabilization. In contrast, ABA-mediated ectopic outgrowth is accompanied by microtubule depolymerization. Therefore, ABA-dependent protrusion formation might be due to a different mechanism from that present in *spr1*, *nek6*, and BPP1 overexpression. The balance between the polymerization and depolymerization of cortical microtubules is essential for proper directional cell expansion, and its disturbance results in the formation of ectopic outgrowths.

ABA-dependent microtubule depolymerization could be attributed to the suppression of microtubule polymerization as in the case of *phs1-1d* mutant. The *phs1-1d* mutation causes the activation of the kinase domain, which phosphorylates α-tubulin to suppress tubulin incorporation into growing microtubules^50^. However, this mechanism is not likely involved in ABA-dependent depolymerization because *phs1-1d* did not enhance ectopic outgrowth. In addition, the total fluorescence of GFP-TUB6 was greater in the ABA-treated cells than control cells, suggesting the involvement of potent destabilization rather than the decrease of tubulin concentration and polymerization competence. When considered together with the observation that short-term ABA treatment did not induce microtubule depolymerization, it seems that long-term ABA treatment may promote the ability to destabilize microtubules through these multiple factors, including the activity of MAPs and the alteration of gene expression and protein levels. In the current study, we are now isolating mutants which exhibit defective ectopic outgrowth under the ABA treatment, and which also exhibit morphological defect similar to those of the microtubule-related mutants. These mutants will provide new insight into ABA-dependent microtubule regulation.

Genetic and physiological analysis suggests that endogenous ABA is required for the promotion of plant growth under the non-stress condition^56^,^57^,^58^,^59^. ABA deficient mutants of *A. thaliana* and tomato show growth suppression in shoots, leaves, and stems^56^,^57^,^58^. The mesophyll cells of *aba1* mutants are significantly smaller than those of the wild type, even under humid conditions^58^. In addition, the overexpression of ABA-responsive *PP2CF1* resulted in hypersensitivity to ABA, accelerated growth of the inflorescence stems, and an increase in cell proliferation and expansion^59^. The ABA-dependent ectopic outgrowth reported here might reflect the role of ABA as a growth-promoting factor.

In summary, we found that ABA promotes ectopic outgrowth in epidermal cells through microtubule depolymerization and reorganization. This function of ABA has not been included among the previously characterized ABA functions: thus our findings will provide a new experimental model for the study of ABA functions in cell growth and morphogenesis.

## Methods

### Plant material and growth conditions

The *Arabidopsis thaliana* Columbia accession was used as the wild type in this study. The mutants of *abi1-1*, *abi2-1*, *abi4-1*, *abi5-1*, *ahg1-1*, *ahg2-1* and *ahg3-1* were described previously^41^,^42^,^43^,^44^,^45^,^46^. The mutants of *ktn1 P393S*, *tua4 S178δ*, *phs1-1*, *spr1-3* and *spr2-2* and GFP-TUB6 line were described previously^8^,^9^,^10^,^11^,^12^,^13^,^49^,^51^. The *gl2-t1* (salk_130214) mutant has a T-DNA insertion in the fourth exon and does not express transcripts for *GL2* as shown in ref. 52. The *aria* mutant (salk_143439C) was obtained from Arabidopsis Biological Resource Center (ABRC) and its genotype is confirmed according to the standard PCR method ( http://signal.salk.edu/tdnaprimers.2.html).

Arabidopsis seeds were surface-sterilized and germinated on the Murashige and Skoog (MS) medium [1/2 MS salt, 1% sucrose (w/v), 0.01% (w/v) myo-inositol, 0.0001% (w/v) pyridoxine hydrochloride, 0.0001% (w/v) nicotinic acid, 0.001% (w/v) thiamine hydrochloride, 0.04% 2-(N-morpholino) ethanesulfonic acid (w/v) and 1% agar]. Plants were grown under a 16 h-light/8 h-dark photoperiod at 23 °C.

In ABA treatment, seeds were germinated on MS medium supplemented with or without 1 μM ABA. The rate of plants with ectopic protrusions was determined after two or three weeks using about 20 plants. The average and standard error was calculated from three independent experiments. During short-term treatment, 7-day old seedlings grown in the absence of ABA were soaked in the liquid MS medium containing ABA and observed under a confocal microscope. In the stress treatment, seedlings were germinated and grown on the MS medium supplemented with 100 mM NaCl. For the etiolation treatment, seedlings were germinated and grown on the MS medium containing 1 μM ABA under the dark condition.

### Microscopy

Seedlings were observed by using stereoscopic microscopes S8AP0 (Leica Microsystems, http://www.leica-microsystems.com/) equipped with a CCD camera (DFC500, Leica) or SMZ1500 (Nikon Instruments, http://www.nikoninstruments.com/) equipped with a digital camera (EOS Kiss X2, Canon, http://www.canon.jp/). Differential interference contrast (DIC) microscopy was conducted by DM5000B (Leica) equipped with CCD camera (DFC500). Confocal imaging was conducted with confocal laser scanning microscopes, FV-1200 (Olympus, http://www.olympus-lifescience.com/) or C1 (Nikon). Images were processed by Image-J software. The quantification of cortical microtubules was conducted according to ref. 7.

In scanning electron microscopy (SEM), plants were fixed in a solution of 1% glutaraldehyde in 50 mM sodium phosphate buffer for overnight, dehydrated in the ethanol series (30, 60, 80, 90, and 100%) and then transferred to a solution of isoamyl acetate. Samples were dried by a critical point dryer (JCPD-5, JEOL, http://www.jeol.co.jp/en/) using liquid CO_2_, coated with gold by a ion sputter (JFC-1200, JEOL) and observed with a scanning electron microscope (JSM-6510LV, JEOL).

## Additional Information

**How to cite this article**: Takatani, S. *et al.* Abscisic acid induces ectopic outgrowth in epidermal cells through cortical microtubule reorganization in *Arabidopsis thaliana*. *Sci. Rep.* **5**, 11364; doi: 10.1038/srep11364 (2015).

## Supplementary Material

Supplementary Information
