# Supplementary material for: Abscisic acid induces ectopic outgrowth in epidermal cells through cortical microtubule reorganization in Arabidopsis thaliana
Source: Sci Rep. 2015 Jun 12;5:11364. doi: 10.1038/srep11364 (PMC4464343; doi:10.1038/srep11364)
Supplement: Supplementary Information [file srep11364-s1.pdf]

Abscisic acid induces ectopic outgrowth in epidermal cells through cortical microtubule reorganization in *Arabidopsis thaliana*

Shogo Takatani, Takashi Hirayama, Takashi Hashimoto, Taku Takahashi & Hiroyasu Motose

Supplemental Figure S1-S5

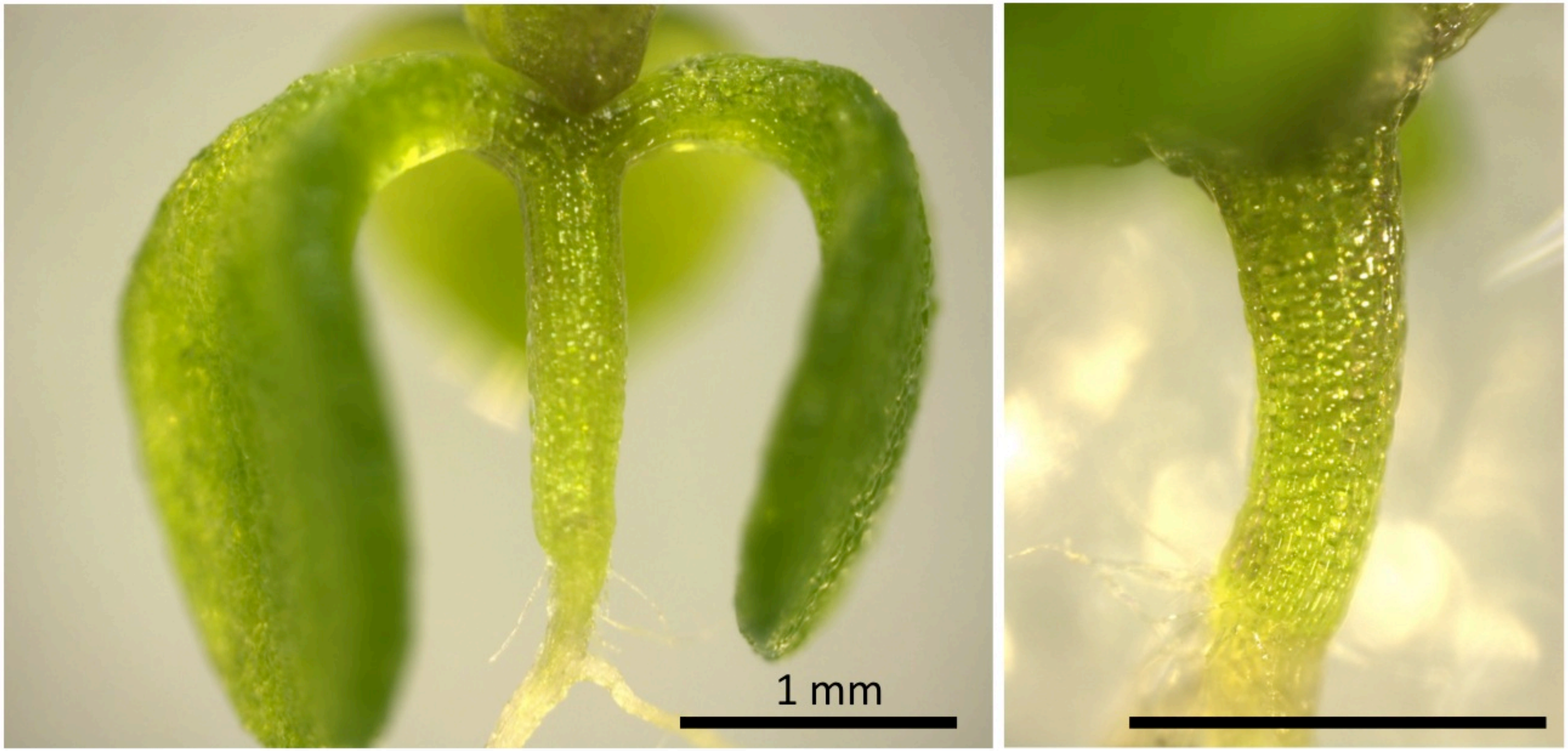

Supplemental Figure S1 | Effect of salt treatment on epidermal morphology. The wild type seedlings were germinated and grown for 3 weeks on medium containing 100 mM NaCl. The ectopic outgrowth was not observed (no protrusions in 20 plants).

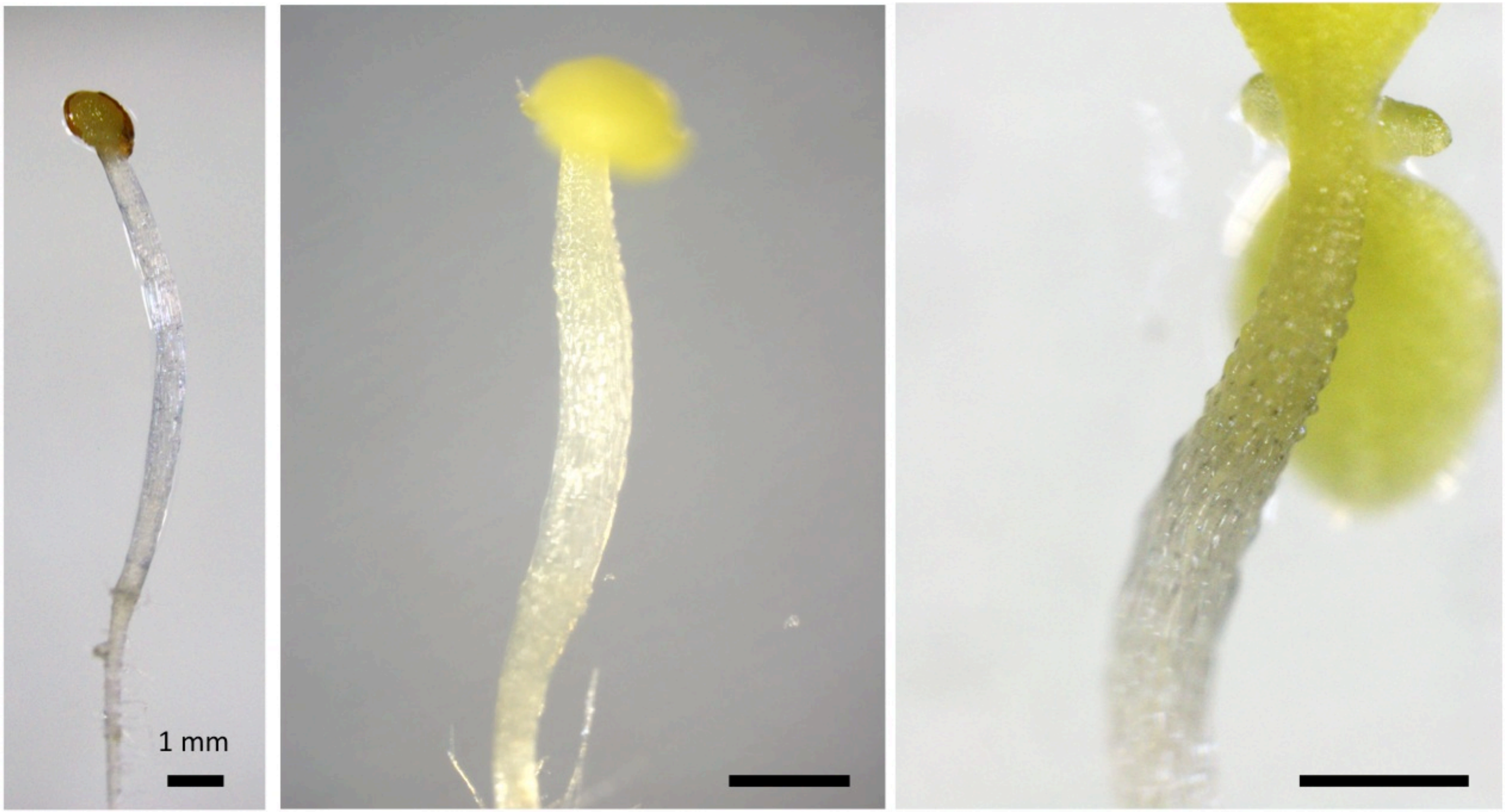

Supplemental Figure S2 | Effect of cell elongation on ABA-induced ectopic outgrowth. The wild type seedlings were germinated and grown for 3 weeks under a dark condition on medium containing 1 μM ABA. The hypocotyl growth was promoted and ectopic outgrowth was suppressed (no protrusions in 15 plants).

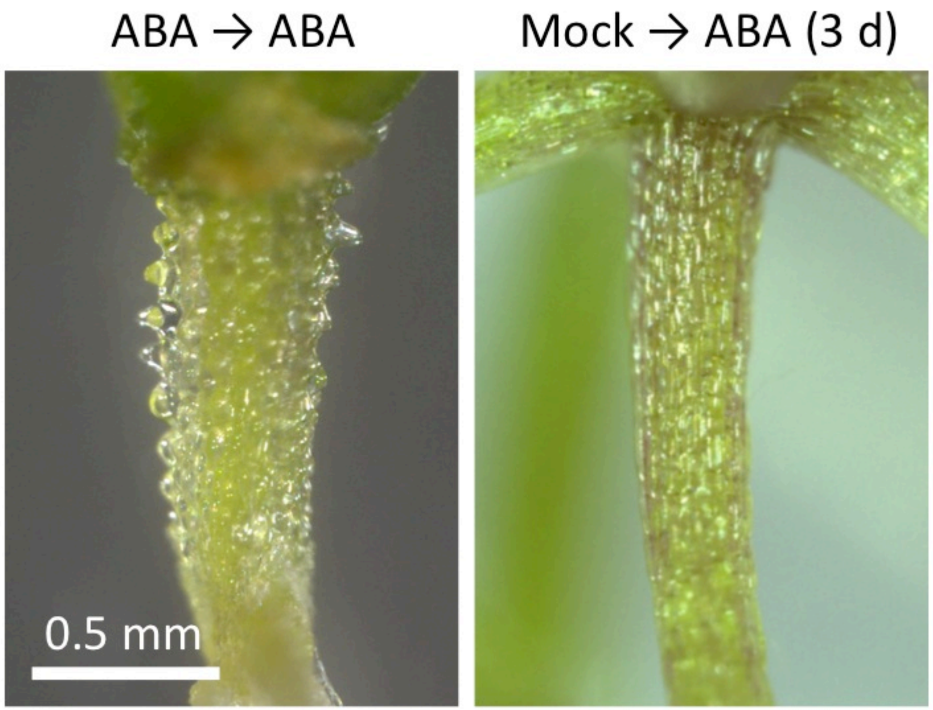

Supplemental Figure S3 | The requirement of ABA for ectopic outgrowth in the early phase of seedling growth. The wild type seedlings were germinated and grown for 3 days on medium without ABA and were transferred to medium containing 1 µM ABA. The morphology of seedlings was observed at 18 days after transfer. Seedlings did not form ectopic protrusions (12 plants).

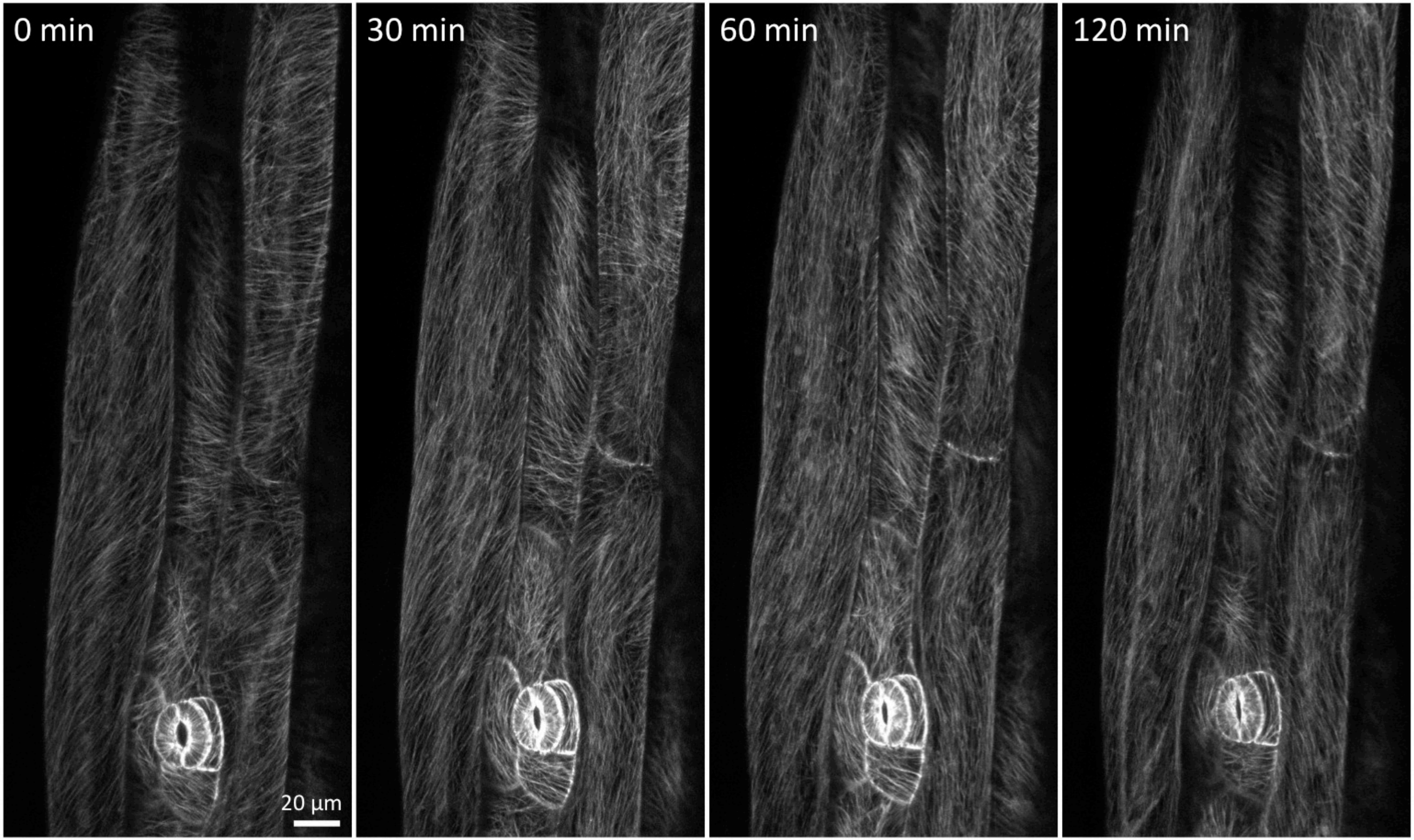

Supplemental Figure S4 | Effect of short-term treatment with ABA on cortical microtubules. The GFP-TUB6 seedlings were grown on medium without ABA, transferred into ABA-containing liquid medium, and observed under a confocal microscopy. ABA treatment promoted the longitudinal orientation of cortical microtubules but not induced disorganizaiton and depolymerization of cortical microtubules.

a

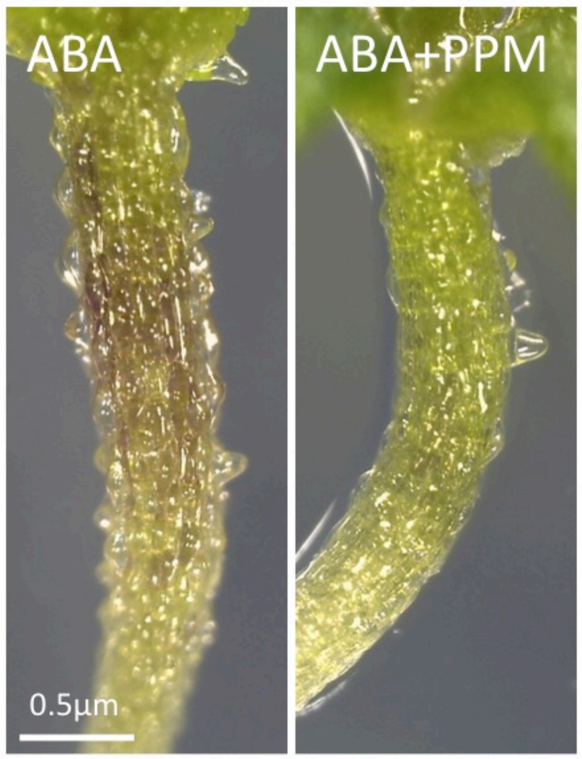

b

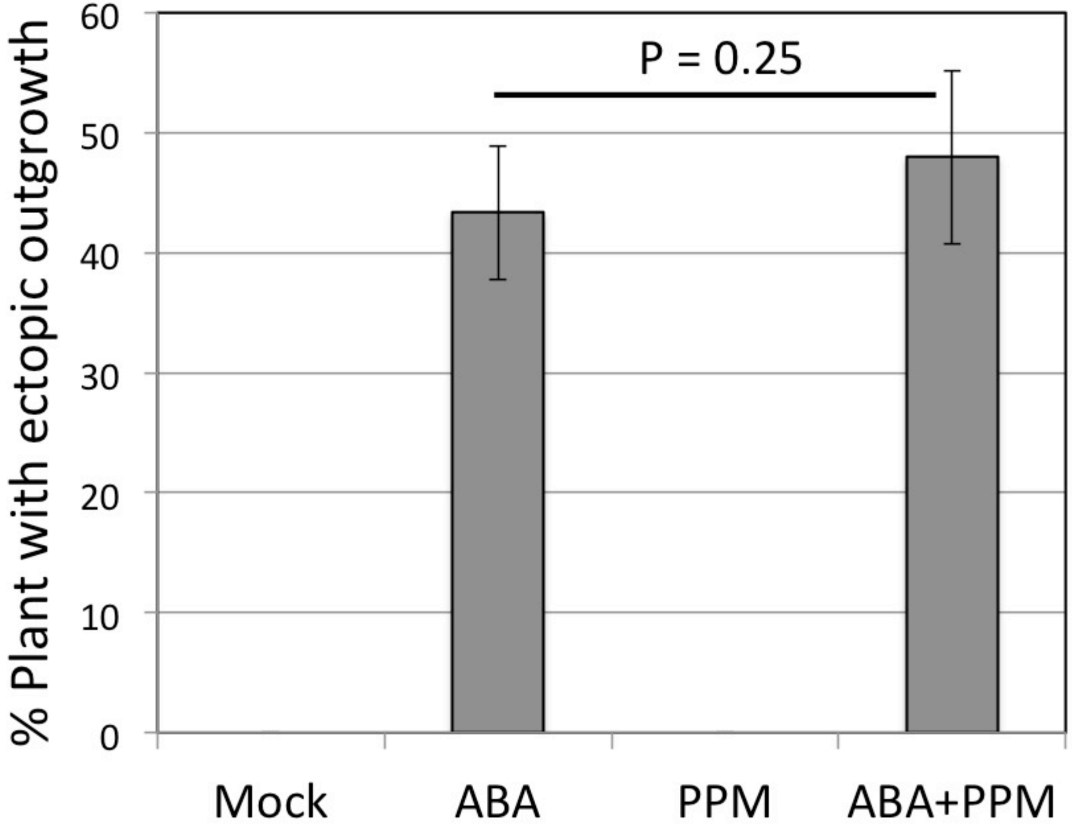

Supplemental Figure S5 | Effect of propyzamide on ABA-induced ectopic outgrowth. The wild type seedlings were germinated and grown for 2 weeks on medium without (Mock) or with 1 μM ABA (ABA), 3 μM propyzamide (PPM) or 1 μM ABA plus 3 μM propyzamide (ABA+PPM). (a) Morphology of hypocotyl epidermal cells. (b) Quantification of ectopic outgrowth. Data are displayed as averages ± SEM of 3 independent experiments. Propyzamide did not significantly affect ABA-induced ectopic outgrowth (P = 0.25 according to the chi-square test for the difference between two proportions).

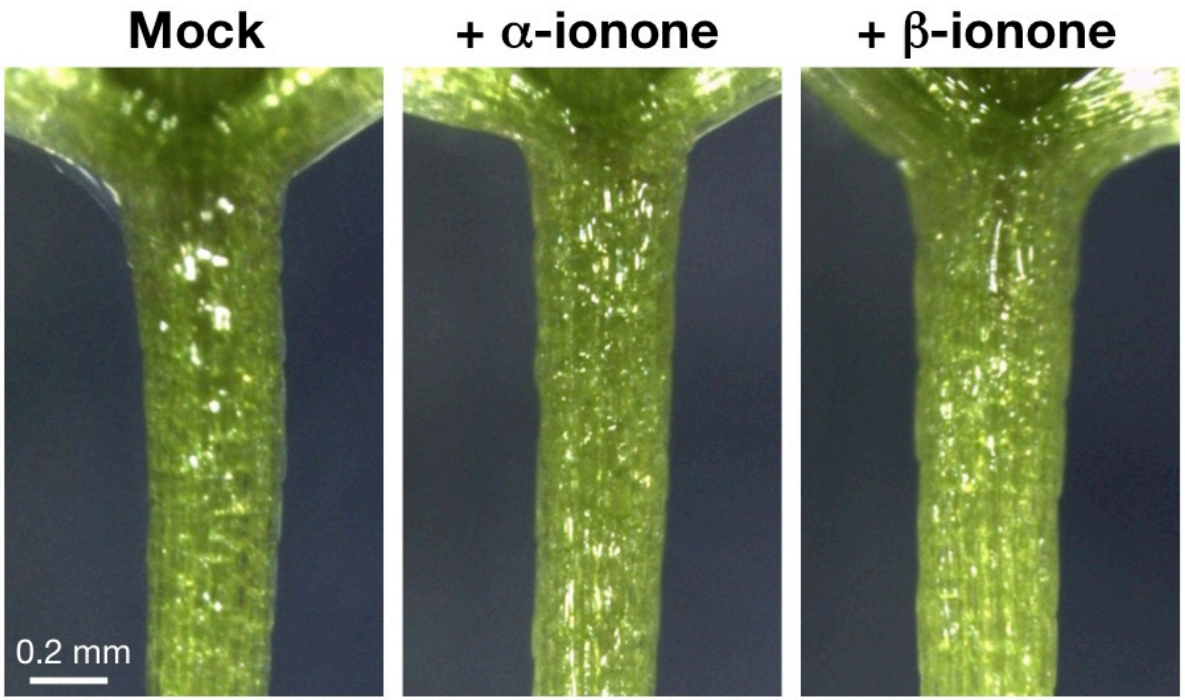

Supplementary information Figure 1 | Effect of α-ionone and β-ionone on the morphology of hypocotyl epidermal cells. The wild type seedlings were germinated and grown for 2 weeks on medium without (Mock) or with α-ionone or β-ionone at a concentration of 1 μM. Ectopic outgrowth was not observed (30 plants in Mock, 38 plants in α-ionone and 20 plants in β-ionone).

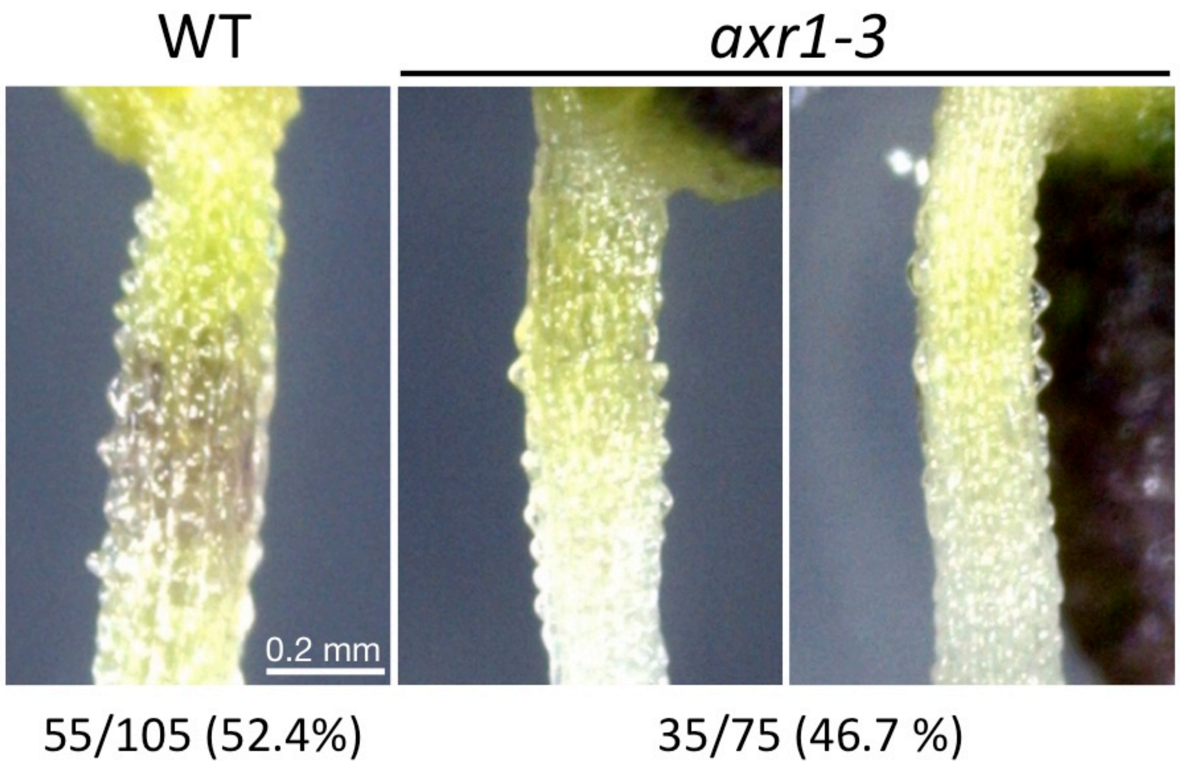

Supplementary information Figure 2 | Effect of ABA on the morphology of hypocotyl epidermal cells in the wild type and *axr1* mutant. Morphology of hypocotyls in the wild type (WT) and *auxin resistant 1-3* (*axr1-3*) mutants grown for 2 weeks in the presence of 1 μM ABA (ABA). The numbers below indicate the numbers of plants with protrusions / total numbers of plants (P = 0.22 according to the chi-square test for the difference between two proportions).
